# The Prevalence of Infectious Keratitis after Keratorefractive Surgery: A Systematic Review and Meta-Analysis Study

**DOI:** 10.1155/2020/6329321

**Published:** 2020-07-28

**Authors:** Shahla Afsharpaiman, Musa Zare, Masoud Yasemi, Tannaz Jamialahmadi, Amirhossein Sahebkar

**Affiliations:** ^1^Health Research Center, Life Style Institute, Bagiyatallah University of Medical Sciences, Tehran, Iran; ^2^Department of Ophthalmology, Jahrom University of Medical Sciences, Jahrom, Iran; ^3^Department of Food Science and Technology, Quchan Branch, Islamic Azad University, Quchan, Iran; ^4^Department of Nutrition, Faculty of Medicine, Mashhad University of Medical Sciences, Mashhad, Iran; ^5^Halal Research Center of IRI, FDA, Tehran, Iran; ^6^Biotechnology Research Center, Pharmaceutical Technology Institute, Mashhad University of Medical Sciences, Mashhad, Iran; ^7^Neurogenic Inflammation Research Center, Mashhad University of Medical Sciences, Mashhad, Iran

## Abstract

**Background:**

The keratorefractive surgeries (KRS) are one of the most common ocular surgeries. One of the dangerous complications of these surgeries is infectious keratitis (IK), which is the second cause of blindness after cataract surgery. The purpose of this study was to estimate the prevalence of IK after KRS in different parts of the world.

**Methods:**

In order to obtain relevant studies, all national and international databases including IranMedex, SID, Magiran, IranDoc, Medlib, ScienceDirect, PubMed, Scopus, Cochrane, Embase, Web of Science, and Google Scholar were searched using standard keywords.

**Results:**

IK prevalence after KRS was 0.000496% (0.000145% for the left eye and 0.000149% for the right eye). IK prevalence after KRS in the United States, Europe, and Asia was 0.000667%, 0.000473%, and 0.000045%, respectively, in all of which the common microorganisms were *Staphylococci*. Meta-regression showed no significant association between IK after KRS and either sample size or publication year of the studies. IK prevalence after KRS in the right eye was more than that in the left one. Also, the probability of IK incidence after LASIK surgery was more than PRK and LASEK. In the evaluation of continents, IK after KRS in the United States was more frequent compared with Europe and Asia.

**Conclusions:**

This study provided data as to the overall prevalence of IK following KRS and its variations according to the types of eye, surgery, pathogenic microorganism, and geographical location.

## 1. Introduction

Keratorefractive surgeries (KRS) including LASIK (laser *in situ* keratomileusis), LASEK (laser-assisted subepithelial keratectomy), and PRK (photorefractive keratectomy) are the most common eye surgeries [1–5]. One of the most important complications of these surgeries is infectious keratitis (IK) [6], which is a leading cause of blindness around the world [7]. IK is an emergency of ophthalmology needing an urgent diagnosis and treatment in order to prevent its irreparable complications [8, 9]. Postoperative keratitis, based on its source, can be infectious or noninfectious [10]. IK can be caused by viral, bacterial, parasitic, and fungal organisms [11]. The most common organisms causing IK are *Staphylococcus aureus*, *Staphylococcus epidermidis*, and *Streptococcus pneumoniae*, which are also present in the normal eye flora [12–20]. Post-LASIK keratitis reports have become increasingly common in recent years [21–34].

The risk factors of IK after KRS include the use of contact lens, persistent or large epithelial defects, and the use of steroid drops. In LASEK, the presence of a loose and manipulated epithelial layer under the contact lens may be another cause of infection [35].

LASIK is, however, the most common refractive surgery method owing to its more advantages compared with PRK to correct ametropia, including faster rehabilitation of vision, reduced stromal damage, decreased irregular astigmatism, least regression, less pain at postoperation, and more ability in the treatment of refractive errors [36–41]. Theoretically, the risk of infection during PRK is higher than that in LASIK, which is because in the PRK method an epithelial defect is created, which is about 6–8 mm and takes approximately 4 days to recover [17]. The rate of infection incidence after LASIK is reported to be between 1 in 1000 and 1 in 5000 [30, 42–45].

Considering the different statistics on the prevalence of IK after KRS in different parts of the world, the present study aimed to cover the lack of a meta-analysis on the evaluation of IK prevalence after KRS worldwide.

## 2. Materials and Methods

### 2.1. Study Protocol

The present study was a systematic review and meta-analysis that aimed to evaluate the prevalence of IK after KRS in different parts of the world.

### 2.2. Search Strategy

In order to find relevant studies, English-language databases including ScienceDirect, PubMed, Scopus, Embase, Web of Science, Google Scholar, and Cochrane as well as Persian-language databases (SID, Magiran, IranMedex, IranDoc, and Medlib) were searched without the time limitation. The search was performed by using the valid and standard keywords “Infectious Keratitis, Keratorefractive Surgery, PRK, LASIK, LASEK, Meta-Analysis” and the Latin equivalents and their mesh. Also, their combinations were searched by using AND and OR operators in English-language bases.

### 2.3. Inclusion and Exclusion Criteria

The inclusion criteria included studies that evaluated the incidence of IK after KRS. The exclusion criteria involved studies with nonrandom sample size, case reports, studies performed on patients with IK, and studies lacking the required information such as the total number of examined eyes or the number of eyes with IK. Low quality of studies based on the STROBE checklist and inaccessible studies were also excluded.

### 2.4. Qualitative Assessment of Studies

The standard international STROBE checklist was used to assess the quality of studies. This checklist contains 22 parts covering different sections of a report, based on which the articles gaining at least a score of 16 were entered to the meta-analysis process.

### 2.5. Data Extraction

Two researchers, independently, performed data extraction from studies to minimize bias in the reports and errors in data collection. The researchers entered the extracted data into a checklist including the name of the researcher, the title of study, the number of patients, the number of afflicted eyes, the incidence of IK in the left and right eyes, the year of the study, and the country and the continent in which the research was conducted. A third researcher checked the extracted data by the two previous researchers to resolve any disagreement.

### 2.6. Statistical Analysis

Due to the fact that some prevalence rates were close to zero or one, for the stability of variances, the double arcsine Freeman–Tukey transformation was used [46]. To examine the heterogeneity of the studies, the Q-Cochrane test and *I*^2^ index (*I*^2^  index is less than 25% of the low heterogeneity, between 25% and 75% of the middle heterogeneity, more than 75% of the high heterogeneity) were used [47]. This study had a high heterogeneity with the *I*^2^ value of 97.2%.

Data were analyzed using STATA software, 14.1 version. Meta-regression was used to show the association between the prevalence of IK after the KRS and the number of samples and the year of the study, and the significance level of the tests (*P* < 0.05) was considered.

## 3. Results

**Figure 1:**
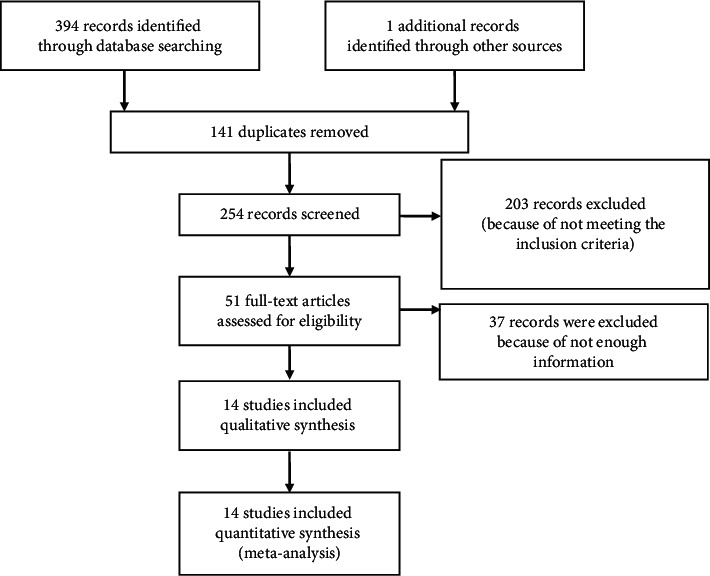
Flowchart of the evaluated studies into meta-analysis.

**Figure 2:**
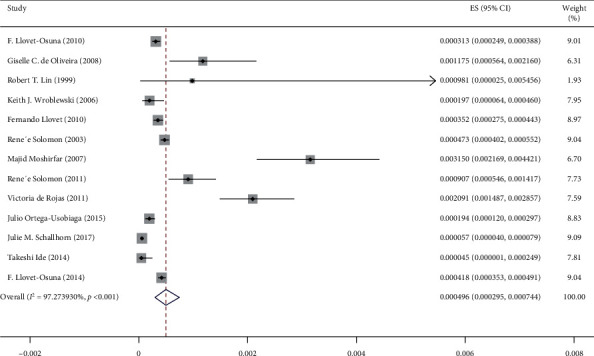
The incidence of infectious keratitis after performing the keratorefractive surgery, according to the author's name and the year of research, based on the random effects model. The midpoint of each line segment reveals the incidence of infectious keratitis after conducting the keratorefractive surgery for each study. The rhombus form also shows the incidence of infectious keratitis after performing the keratorefractive surgery for all studies.

The entry steps of studies into the process of systematic review and meta-analysis are presented in Figure 1. Through 14 examined studies with a sample size of 2,018,558 eyes, the prevalence of IK after KRS was estimated to be 0.0005% (95% confidence interval: 0.0002%–0.0008%). One study performed in 2007 in Iran [48] was considered as irrelevant. Therefore, with the elimination of this study, the prevalence of IK after surgery, again, was calculated, and the rate of 0.0004% (95% interval of confidence: 0.0002%–0.0007%) was obtained (Figure 2).

The study information that entered into the process of systematic review and meta-analysis after the qualitative assessment process is presented in Table 1.

In this meta-analysis, in addition to the estimation of the total prevalence of IK after KRS, the incidence of IK was evaluated based on different subtypes such as countries, continents, and direction (left or right) of the examined eyes. The results are shown in Table 2.

The prevalence of IK was 0.000145% in the left eye and 0.000149% in the right eye, after the KRS. The incidence of IK after LASIK, LASEK, and PRK was 0.000554%, 0.000046%, and 0.000122%, respectively.

In the analysis of different countries, the incidence of IK after KRS in Japan was 0.000045%, in Spain 0.000473%, in the United States 0.000594%, and Brazil 0.001175%. In the analysis of the continents, the incidence of IK after KRS in America, Europe, and Asia was 0.000667%, 0.000473%, and 0.000045%, respectively (Table 2).

In terms of microorganisms after KRS, the prevalence of the fungi was 0.000041%, MRSA 0.000093%, staphylococci (except MRSA) 0.000142%, and viridans streptococci 0.000001% (Table 2).

**Figure 3:**
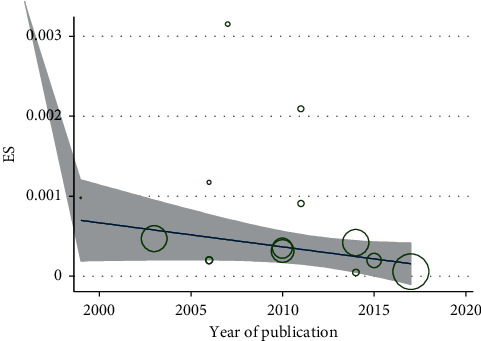
Relationship between the prevalence of infectious keratitis after performing the keratorefractive surgery with the year of publication of the article by using the meta-regression model.

**Figure 4:**
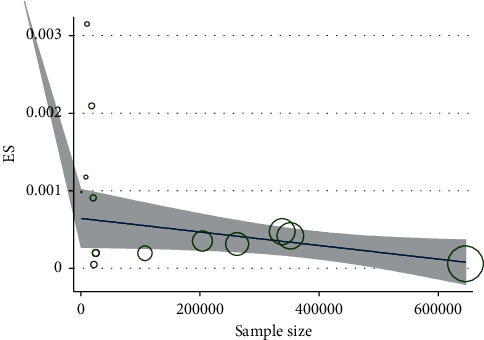
Relationship between the incidence of infectious keratitis and the number of research samples after keratorefractive surgery.

Based on Figures 3 and 4, there was no significant association between the incidence of IK after performing KRS with the year of publication of the study and the number of research samples (*P* > 0.05).

## 4. Discussion

The prevalence of infectious keratitis was 0.0004% after keratorefractive surgery based on the evaluated studies published between 1999 and 2017. Thus, the risk of infectious keratitis after keratorefractive surgery was about 4 per 10,000 eyes, which can be considered as rare. In the study of the prevalence of infectious keratitis conducted on each eye, the prevalence in the right eye was slightly more than that in the left one. Generally, in the KRS, the preoperative process of periocular and ocular prepping is carried out simultaneously on both eyes, and the surgery is frequently performed on the right side first. Despite that, depending on the surgeon and other factors such as significant refractive error difference between the eyes, surgery may be started on either the right or the left eye. Due to the passing of time and probability of not observing the complete sterility process of the replacement set, infection may occur more frequently in the second eye.

A comparison of the studies carried out in different countries revealed that the highest incidence of infectious keratitis after KRS was reported from Brazil, the United States, Spain, and Japan, respectively. In continental studies, the highest prevalence of this keratitis was in America and the lowest prevalence was in Asia. It is noted that there was no study conducted in Africa. The highest incidence of infectious keratitis after keratorefractive surgery in the continent of America was 1.4 and 14 times more than those of Europe and Asia. Possible reasons for the higher prevalence of postrefractive surgery infections in America compared with other continents are the higher number of refractive surgery centers and, subsequently, more reports of these infections. These factors can therefore serve as bias regardless of the racial diversity. On the other hand, in Asia, especially in the developing countries, the number of refractive surgery centers is relatively low, resulting in less reports of infection prevalence.

Based on the study results, the incidence rate of infectious keratitis after the operation of LASIK, PRK, and LASEK was 0.000554%, 0.000122%, and 0.000046%, respectively. According to the previous studies, the incidence of keratitis was reported between 0.02% and 0.8% after PRK [49–61] and 0% and 1.5% after LASIK [62]. Another study reported that the incidence of infectious keratitis after PRK was estimated as one in every 1000 cases, and after LASIK, one in every 5000 cases [63]. In another study, bacterial keratitis after PRK was reported as a rare complication, and its prevalence was reported to range between 1/1000 and 1/5000 [61, 64]. Although, theoretically, due to epithelial defect, it seems that the keratitis incidence following PRK and LASEK should be higher than LASIK, our results showed that the incidence after LASIK was 4.5 times more than PRK, and about 12 times more than LASEK surgery. This finding could possibly be attributed to the manipulation of more corneal tissue in order to create a flap and the need for more tools during LASIK, which increases the risk of infection. It must also be noted that these studies could be biased as the higher incidence of infectious cases in LASIK could simply be related to the fact that this surgery is more common than the other types. Moreover, in LASIK, corneal flap may be created with a microkeratome or femtosecond laser. Based on different studies, the prevalence of infection is higher with a microkeratome because of more manipulation and higher possibility of inducing corneal epithelial defect. The method of creating a flap is thus important in LASIK surgery [65]. Unfortunately, data on this point were not provided in the majority of studies, thereby precluding the possibility of its consideration in the present analysis.

In our study, the most common microorganisms responsible for IK were staphylococci (except MRSA), MRSA, fungi, *Staphylococcus aureus*, and *Streptococcus hemo viridans*, respectively. Therefore, the most common microorganism was from the *Staphylococcus* strain. The incidence of fungal keratitis in the world was between 17% and 36% in cases of corneal ulcers, with an estimation of 44%–47% in India [66–69]. In the three-year study of Bharathi et al., among all types of keratitis, 34.4% of the people were reported with fungal keratitis [70]. In the study by Diaz-Valle et al., 37.23% of cases of corneal ulcers were due to fungal keratitis [71]. In the study by Garg et al. [72], the microorganisms that caused IK after LASIK were fungi (4 eyes), *Nocardia asteroides* (5 eyes), atypical mycobacteria (4 eyes), Acanthameba (2 eyes), *Corynebacterium* (1 eye), and *Staphylococcus epidermidis* (1 eye). Elsewhere, the reported microorganisms responsible for IK after the LASIK operation were *Mycobacterium* [14, 73], fungi [24, 74], *Nocardia* [30, 75], *S. aureus* [76, 77], *S. viridans* [78, 79], coagulase-negative staphylococci [17, 21], and *S. pneumoniae* [22, 80]. It is important to note that after KRS, the bandage soft contact lens is often used for about 5–7 days depending on the wound healing process, but these lenses can increase the risk of IK by about 5- to 20-folds more [81, 82]. In a previous study [19], the rate of contamination of soft contact lens used in refractive imperfections surgery was 18.3%, and the only isolated microorganism was *S. epidermidis*. Also, in some other studies [83–86] conducted on the contamination during eye surgery, the *S. epidermidis* was the main reported microorganism. Therefore, until the corneal epithelial defect is healed, regular and frequent corneal examination is needed and bandage contact lens should be removed as soon as possible.

The meta-regression analysis also showed that there was no significant correlation between the incidence of IK after keratorefractive surgery and the publication year of the study (*P*=0.832). We observed that, in recent years, the prevalence of IK declined after keratorefractive surgery, but this decreasing trend was not statistically significant (Figure 3). Likewise, there was no significant correlation between the prevalence of IK after keratorefractive surgery and the number of research samples (*P*=0.801). Although with the increase in the number of samples, the incidence of infectious keratitis after keratorefractive surgery was numerically decreased, this decrease was not statistically significant (Figure 4).

## 5. Study Limitations

The present study was limited in a number of ways including the lack of uniform distribution of the evaluated studies in different countries and continents, the lack of a study in this regard in some continents such as Africa and the failure to report the incidence of IK after KRS in this continent, and the lack of stratified analysis in terms of age group due to the fact that some studies did not mention the age group. Moreover, the study analysis was not carried out based on the type of antibiotic received by the patients because only a small number of studies referred to the antibiotics used by the patients.

## 6. Conclusion

According to the results of the current meta-analysis, the prevalence of IK after KRS was slightly higher in the right eye than the left eye. In terms of the microorganisms involved in the development of postoperative IK, the most common microorganisms were *Staphylococcus* strains and other microorganisms had a lower contribution to this infection. Also, the likelihood of IK after LASIK was higher than PRK and for PRK was more than that of LASEK. Therefore, care should be taken with the type of surgery and selection of prevention methods to reduce the occurrence of IK as well as arrangement for more visits of patients after surgery. In the evaluation of countries, the highest and lowest incidences of IK after KRS were observed in Brazil and Japan, respectively. With respect to continents, the highest and lowest incidences of IK after KRS were found in the America and Asia, respectively, though this finding might have been biased by more reports of the infection from the former.

## Figures and Tables

**Table 1:** Extracted data from articles that are included in the meta-analysis.

Reference	Author	Year of study	Year of publication	Country	Continent	Age group (years)	Number of total eyes	Number of eyes that have infectious keratitis
[49]	F. Llovet-Osuna	2002–2009	2010	Spain	Europe	37.5 (19–62)	262191	82
[50]	Giselle C. De Oliveira	1997–2002	2006	Brazil	South America	30.33	8508	10
[44]	Robert T. Lin	1997	1999	United States of America	North America	—	1019	1
[51]	Keith J. Wroblewski	1995–2004	2006	United States of America	North America	—	25337	5
[52]	Fernando Llovet	2002–2008	2010	Spain	Europe	35.5	204586	72
[53]	Rene´e Solomon	2001	2003	United States of America	North America	—	338550	160
[54]	Majid Moshirfar	1996–2004	2007	United States of America	North America	—	10477	33
[55]	Rene´e Solomon	2008	2011	United States of America	North America	—	20941	19
[56]	Victoria De Rojas	2003–2009	2011	Spain	Europe	38.1 (25–64)	18651	39
[57]	Julio Ortega-Usobiaga	2010–2013	2015	Spain	Europe	21–70	108014	21
[58]	Julie M. Schallhorn	2008–2015	2017	United States of America	North America	—	645957	37
[59]	Takeshi Ide	2007–2007	2014	Japan	Asia	—	22415	1
[60]	F. Llovet-Osuna	—	2014	Spain	Europe	—	351712	147
[48]	Sepehr Feizi	—	2007	Iran	Asia	28.2 (19–49)	200	49

**Table 2:** Results of the meta-analysis of articles.

Subgroups		Number of studies	Prevalence of infectious keratitis (%)	Low	Up	*P* value	*I* ^2^ (%)	*Z*
Overall		13	0.000496	0.000295	0.000744	<0.001	97.2	7.448384

Country	Brazil	1	0.001175	0.000564	0.002160	—	—	5.480057
	Spain	5	0.000473	0.000294	0.000693	<0.001	94.2	8.603066
	USA	6	0.000594	0.000173	0.001221	<0.001	98.2	3.753000
	Japan	1	0.000045	0.000001	0.000249	—	—	1.414219

Continent	America	7	0.000667	0.000241	0.001275	<0.001	98	4.301790
	Europe	5	0.000473	0.000294	0.000693	<0.001	94.2	8.603066
	Asia	1	0.000045	0.000001	0.000249	—	—	1.414219

Surgery	LASIK	6	0.000554	0.000331	0.000832	<0.001	9.531e + 01%	7.913473
	LASEK	1	0.000046	0.000031	0.000067	—	—	9.295248
	PRK	3	0.000122	0.000063	0.000197	0.347189	5.4	6.122264

Eye	Right	3	0.000149	0.000118	0.000182	0.488585	0	16.814513
	Left	3	0.000145	0.000096	0.000204	0.064426	63.5	9.701854

Organism	Fungal	3	0.000041	0.000000	0.000157	0.064144	63.5	1.585417
	Methicillin-resistant (MRSA)	3	0.000093	0.000018	0.000211	0.313975	13.6	3.119117
	*Staphylococci*	3	0.000142	0.000007	0.000409	0.000760	86	2.360040
	*Staphylococcus aureus*	5	0.000015	0.000000	0.000069	0.003517	74.4	1.288479
	*Streptococcus hemo viridans*	3	0.000001	0.000000	0.000017	0.218840	34.1	0.635427
